# Strategies for Pharmacological Organoprotection during Extracorporeal Circulation Targeting Ischemia-Reperfusion Injury

**DOI:** 10.3389/fphar.2015.00296

**Published:** 2015-12-22

**Authors:** Aida Salameh, Stefan Dhein

**Affiliations:** ^1^Clinic for Pediatric Cardiology, Heart Centre University of LeipzigLeipzig, Germany; ^2^Rudolf-Boehm-Institute for Pharmacology and Toxicology, University of LeipzigLeipzig, Germany

**Keywords:** cardiopulmonary bypass, organoprotection, PARP, MMP, MPT, antioxidants, cardioplegia

## Abstract

Surgical correction of congenital cardiac malformations or aortocoronary bypass surgery in many cases implies the use of cardiopulmonary-bypass (CPB). However, a possible negative impact of CPB on internal organs such as brain, kidney, lung and liver cannot be neglected. In general, CPB initiates a systemic inflammatory response (SIRS) which is presumably caused by contact of blood components with the surface of CPB tubing. Moreover, during CPB the heart typically undergoes a period of cold ischemia, and the other peripheral organs a global low flow hypoperfusion. As a result, a plethora of pro-inflammatory mediators and cytokines is released activating different biochemical pathways, which finally may result in the occurrence of microthrombosis, microemboli, in depletion of coagulation factors and haemorrhagic diathesis besides typical ischemia-reperfusion injuries. In our review we will focus on possible pharmacological interventions in patients to decrease negative effects of CPB and to improve post-operative outcome with regard to heart and other organs like brain, kidney, or lung.

## Introduction

At the beginning of the twentieth century most of the inborn cardiac malformations could not be operated due to the absence of a medical device for body perfusion during open heart surgery.

In 1885 two physicians invented a “respiration system” which could be considered as a first prototype of a heart-lung machine: Maximilian von Frey and his colleague Max Gruber both working at the Carl-Ludwig Institute in Leipzig developed a machine capable of perfusing and oxygenating isolated organs (von Frey and Gruber, 1885). However, this machine was intended to be used for basic research and not for open heart operations. Nearly, 70 years later in 1954 Gibbon designed a device which was able to bypass heart and lung and he also was the first cardiac surgeon who used it during open heart operations (Gibbon, 1954). This heart-lung machine -the so-called cardiopulmonary bypass (CPB)—was a milestone in cardiac surgery together with the development of surface hypothermia by Bigelow (1954). Both medical developments were a tremendous step for heart operations and the onset of our modern cardiac surgery. Although the heart-lung machine has now become indispensable and allows very complex heart operations such as the correction of inborn cardiac diseases like Morbus Fallot or the hypoplastic left heart syndrome, the potential adverse effects of this technique on sensitive organs like brain or kidney cannot be ignored. In particular, in children there are several reports on negative effects of the cardiopulmonary bypass (CPB) on the developing brain with some of the cerebral areas including the hippocampus being especially sensitive to ischemia and reperfusion injury (Chugani, 1998). It is a well-known fact that lesions in the hippocampus, which is involved in learning and memory processes, might lead to cognitive impairments like learning disabilities, memory deficits, behavioral disorders, and hyperactivity (Su and Undar, 2010). Despite the fact that neurological assessment is inherently difficult in neonates and small children there are several lines of evidence that the potential adverse effects of CPB on the developing brain are subtle and that neurological deficits manifest themselves often years after successful surgical correction (Pua and Bissonnette, 1998). In fact, there are some clinical studies on children with complex cardiac malformations operated in early childhood, which showed that CPB might have negative implications on the later neurological outcome (Calderon et al., 2010; Bellinger et al., 2011).

The flow generated by the heart-lung machine usually has a constant and mostly laminar flow profile. This is in contrast to our own cardiovascular system which produces a pulsatile blood flow. This should not have a negative impact on cerebral perfusion as -due to autoregulative processes (Bayliss-effect)—cerebral perfusion remains constant within a wide pressure range (50–150 mmHg) (Bayliss, 1902). However, there is some evidence that a more physiological pulsatile flow might be superior over the commonly used non-pulsatile flow during CPB (Kusch et al., 2001). Other organs like kidney, liver, and intestine are less tightly autoregulated, and thus also possibly affected by CPB, especially in young organisms (Tirilomis et al., 2009, 2013; Doguet et al., 2012).

However, more than two-thirds of all cardiac surgical procedures do not involve inborn cardiac malformations in the young but aortocoronary bypass grafts and valve replacements in elderly patients. These patients also have a substantial probability to develop severe impairments like neurological complications (disorientation, transient ischemic attacks, and stroke), renal failure with the necessity of dialysis, or lung disorders with prolonged artificial ventilation (Alexander et al., 2000; Boldt et al., 2003; Reis Miranda et al., 2005; Kamiya et al., 2014). It should be mentioned that stroke and TIA may be attributed to clots embolizing the A. cerebri media area and disorientation probably is the consequence of global low flow ischemia affecting the water-shed region of hippocampus.

It seems to be remarkable that despite an appropriate “cardiac output” delivered by the heart-lung-machine and body cooling, organ injuries develop, which impair the post-operative result. Moreover, both bypassed organs -heart and lung- are burdened with ischemia and reperfusion injury, which also has a negative impact on the post-operative outcome (Royster, 1993; Slottosch et al., 2014).

In general, CPB initiates a systemic inflammatory response (SIRS) which is presumably caused by contact of blood components with the surface of CPB tubing. As a result, leucocytes, monocytes, endothelial cells, platelets and the complement system are activated, which subsequently lead to a release of a plethora of pro-inflammatory mediators and cytokines. The activation of different biochemical pathways may result in the occurrence of microthrombosis, microemboli, in depletion of coagulation factors and haemorrhagic diathesis (Murphy and Angelini, 2004).

On the cellular level ischemia and reperfusion induce several signaling cascades including translocation of numerous transcription factors [for example: HIF1α (hypoxia-induced factor 1α) or AIF (apoptosis inducing factor)], induction of caspase pathway, activation of matrix-metalloproteinases (MMPs), PARP-activation (poly-ADP-ribose polymerase), and formation of reactive oxygen and nitrogen species (Salvesen, 2002; Ye et al., 2002; Yu et al., 2006; Smith et al., 2008; Lange et al., 2010). These signaling cascades are involved in regulatory mechanism of cell survival and cell death finally leading to potential organ dysfunction.

In the past decades several protecting strategies have been applied to ensure greater safety during CPB: bubble traps and screen filters to reduce the risk of embolization, lung protective mechanical ventilation to avoid respiratory distress syndromes, improved pH, temperature and fluid management, and a pulsatile flow (which has been shown to improve renal function and regional oxygen saturation of the brain) or pharmacological approaches to minimize organ damage (Zhao et al., 2011; De Somer, 2013; Ferrando et al., 2015; Nam et al., 2015). Various drugs have been tested -mostly in various animal models- to reduce organ malfunction: drugs targeting the Na^+^/H^+^-exchanger or the I_*KATP*_ channel, inhibitors of MMPs, PARP-inhibitors, antioxidants, guanylyl cyclase stimulators, or anti-inflammatory drugs.

In our review we will focus on possible pharmacological interventions in patients to decrease negative effects of CPB and to improve post-operative outcome with regard to heart and other organs like brain, kidney, or lung.

### PARP-inhibitors

DNA-strand breaks mainly occur during the reperfusion phase when oxygen levels increase. Reactive oxygen species (ROS) and peroxynitrite (which is formed in the presence of NO) diffuse into the nucleus and induce disruption of the genetic material. The resulting single strand DNA nicks are detected by PARP and labeled with PAR-chains. These in turn are a signal for other DNA repairing enzymes such as DNA ligase and DNA polymerase beta (Isabelle et al., 2010). After the repair PAR-chains are degraded. Over-activation of PARP depletes NAD^+^ (nicotinamide adenine dinucleotide) stores and finally leads to a reduction of ATP-levels, which at the end is disastrous for the cell. Moreover, PAR-chains cause release of AIF (apoptosis-inducing factor) from the mitochondria into the cytoplasm. AIF then translocates into the nucleus and initiates the so-called parthanatos, which is irreversible and results in cell death (Wang et al., 2009).

Inhibitors of the PARP-pathway such as minocycline might prevent from ATP-depletion and might maintain intracellular ATP-content. Minocycline is a broad spectrum antibiotic attributed to tetracycline class. It has bacteriostatic effects and is frequently used for treatment of acne vulgaris or Lyme's disease. Moreover, it has neuroprotective and anti-inflammatory properties (Giuliani et al., 2005; Kim and Suh, 2009). In a rat model of hypothermic cardiac arrest Drabek et al. (2014) could demonstrate that minocycline reduced ischemia-induced elevated TNFα-levels in the brain (Drabek et al., 2014). The same effect could be shown in liver, where minocycline significantly decreased hepatic TNFα and IL-1β expression and improved liver function (Li et al., 2015). The PARP-inhibitory effect of minocycline and its positive influence on cell apoptosis were also revealed in other studies and by our working group: piglets subjected to cardio-pulmonary bypass showed less organ dysfunction (hippocampus, liver, kidney) when minocycline was administered before bypass and during reperfusion (Tao et al., 2010; Dhein et al., 2015; Salameh et al., 2015).

The typical plasma concentration in patients receiving minocycline treatment for medical reasons is in the range of 1–2 μmol/L (Sakellari et al., 2000). This is more than ten times higher than the reported concentration needed for sufficient PARP-inhibition (Alano et al., 2006). It seems reasonable to consider minocycline application during cardio-pulmonary bypass in clinical settings, but at the moment patient studies to corroborate the protective effects of minocycline are missing. Another tetracycline with a similar chemical structure as minocycline is doxycycline and there is evidence that this tetracycline derivate also has cytoprotective activities. According to a study of Schwartz et al. (2013) the cytoprotective effects of both tetracycline derivatives are exclusively confined to them, as other derivatives tested did not show any positive effects in ischemia/reperfusion injury. The mechanism by which the protection is obtained is not very clear but it was proposed that inhibition of the MPT (mitochondrial permeability transition) pores is responsible for improved cell survival. Another interesting feature of doxycycline is its activity against matrix metalloproteinases (MMPs), which is described below.

A wide range of other PARP-inhibitors has been used mainly in basic research. In animal or cellular models 3-aminobenzamide, PJ34, or INO-1001 have been tested in ischemia and reperfusion and it has been found that these pharmaceuticals are very effective in blocking PARP. However, until now large clinical trials have not been carried out.

3-aminobenzamide was among the first PARP-inhibitors and was described in the eighties to inhibit PARP by about 90% in an *in vitro* assay (Purnell and Whish, 1980). In the following years 3-aminopenzamide was used in experimental models of stroke, renal ischemia, myocardial infarction, or heart transplantation and it was found that in all cases application of 3-aminobenzamide prior to cellular injury led to a better outcome of the examined organs (Chatterjee et al., 2000; Liaudet et al., 2001; Fiorillo et al., 2003; Koh et al., 2005). In a more recent study 3-aminobenzamide was tested in a rabbit model of CPB (Yeh et al., 2006). The authors could demonstrate that PARP-inhibition resulted in a significant decrease in cytokine levels and as a consequence a reduction of ROS, which in turn led to less apoptosis of cardiomyocytes and to improved contractile function of the hearts.

Another well-known PARP-inhibitor is PJ34. It is classified as a phenanthridinone and besides its PARP-inhibiting activity it exhibits also antiviral and anti-tumorous functions (Lin et al., 2014; Liang et al., 2015). Additionally, Szabo et al. (2004a,b) and Andrasi et al. (2005), demonstrated in a canine model of CPB that administration of PJ34 was able to significantly reduce endothelial dysfunction of mesenteric vessels (frequently seen after CPB). Moreover, it improved myocardial contraction and even pulmonary function.

A similar profile of action is attributed to INO-1001, which showed cardioprotective and lung protective properties in a canine model of cardiopulmonary bypass injury and also seemed to diminish mesenteric dysfunction as described by the same working group (Szabo et al., 2004c,d). In another study of Dhein et al. (2008) piglets of up to 10 kg were examined in a model of extracorporeal circulation and it was found that the post-ischemic elevated TNFα (tumor necrosis factor α)- and HSP70 (heat shock protein 70)-levels as well as lung impairment were significantly reduced by INO-1001 pre-treatment. Because of promising results in basic research a clinical trial was initiated involving patients with acute myocardial infarction undergoing percutaneous transluminal coronary angioplasty (Ekblad et al., 2013). In these patients INO-1001 seemed to decrease inflammatory reactions during recovery. However, frequently seen non-serious adverse events were described: elevation of liver enzymes, nausea, vomiting, and increased QTc (corrected QT-time; Morrow et al., 2009). This might be the reason why more detailed investigations on possibly protective effects of INO-1001 on the heart during ischemia and reperfusion were not continued in patients.

While in anti-cancer therapy PARP-inhibitors are still investigated in ongoing studies there are so far no human studies in organ protection during CPB. This might be due to the unfavorable side effects.

### MMP-inhibitors

A collagenolytic factor was first discovered 1962 by Gross and Lapiere when they investigated how tadpoles lose their tails during metamorphosis (Gross and Lapiere, 1962). This factor was later named MMP-1 and to date more than 20 different MMPs are discovered. The MMPs are zinc-dependent endoproteinases involved in the degradation of extracellular matrix, in the cleavage of cell surface receptors and in the regulation of proliferation, cell apoptosis and migration. Moreover, they influence cytokine processing for example activation of TNFα or IL-1β, thereby modulating leucocyte recruitment and inflammatory processes (Van Lint and Libert, 2007). A variety of cardiovascular diseases are related to a dysfunction of MMPs: the development of aortic aneurysms which was ascribed to a MMP-17 deficiency, or an over activation of MMP-2, plaque vulnerability in patients with angina pectoris, as well as impaired wound healing in patients suffering from diabetes mellitus (Chen et al., 2015b; Martin-Alonso et al., 2015; Uccioli et al., 2015; Wang et al., 2015). Opponents of the MMPs are the TIMPs (tissue inhibitors of metalloproteinases) from which four different inhibitors are known. Interestingly, very recently in a meta-analysis of thoracic aortic aneurysms the expression of MMP-2, MMP-9 and the relation of these proteinases to TIMP-1 and TIMP-2 were analyzed and it was found that thoracic aortic aneurysms with bicuspid aortic valves had a different expression pattern of MMPs and TIMPs compared to aortic aneurysms with tricuspid aortic valves (Rabkin, 2014). Since, MMPs also play a role in inflammation and cytokine production it was not far to seek for a role of MMPs during CPB. Indeed, it was detected that after CPB plasma MMP-9 levels were significantly elevated reaching control levels after about 24 h. As a consequence leucocyte counts were also increased. However, as described in the patient trial of Lin et al. (2015) this was not associated with bypass time or pulmonary dysfunction. In contrast, another patient trial showed that elevated MMP-8 and MMP-9 levels are associated with impaired oxygenation and pulmonary dysfunction after CPB (Beer et al., 2015). Thus it may be of interest to inhibit MMPs during CPB.

As mentioned above some of the tetracyclines are potent MMP-inhibitors, these are minocycline, doxycycline and oxytetracycline (Schwartz et al., 2013). In some animal studies doxycycline was very effective in reducing MMP-9-, TNFα-, and IL-1β-levels and it also showed protective effects on lung damage after CPB (Wang et al., 2014; Zhang et al., 2014). Decreased TNFα-levels in the brain were also found by Drabek et al. (2014), attributed to minocycline application prior to hypothermic cardiac arrest in rats. Thus, one could conclude that this also might be due to MMP-inhibition by minocycline although in their study MMP-levels have not been measured. Another mechanism was proposed by Schwartz et al. (2013), who demonstrated in a study on hepatocytes exposed to simulated ischemia and reperfusion that minocycline and doxycycline inhibited cellular death by suppression of MPT and not by MMP-inhibition.

Most of the MMP-inhibitors have only been examined in animal models as they are not approved for human medical treatment. Thus, despite extensive pre-clinical research up to date there is no MMP-inhibitor tested in clinical trials which holds promise for effective medication in patients.

In summary, blocking of MMPs is a very useful tool in organoprotection during CPB, but until now there is no MMP-inhibitor “in the pipeline” to effectively protect organs during CPB in a clinical setting. Since, doxycycline, minocycline, and oxycycline are approved drugs—although the application of these medicaments during CPB would be an “off-label use”—it might be worth to set up clinical trials to investigate the potential of this medication during CPB.

### Inhibitor of Na^+^/H^+^-exchanger (NHE)

Ischemic or hypoxic conditions lead to intracellular acidosis, resulting in an activation of several transmembrane ion pumps with the aim to regain normal intracellular pH. The following membrane transporters are involved in this process: intracellular acidification activates the NHE which exchanges H^+^ ions against Na^+^. This would increase intracellular pH but finally will lead to an undesirable increase in intracellular sodium. Additionally, due to a dropdown in ATP, Na^+^/K^+^-ATPase (which under normal conditions maintains transmembrane sodium and potassium gradients) is inhibited which also contributes to the elevated sodium levels. Furthermore, low pH activates the Na^+^/${\text{HCO}}_{3}^{-}$ symporter, which carries NaHCO_3_ into the cell to buffer excess H^+^ ions (Lagadic-Gossmann et al., 1992). This process also leads to an accumulation of intracellular Na^+^ which at the end stimulates the Na^+^/Ca^2+^ exchanger to extrude sodium against calcium. Enhanced intracellular calcium levels on the other hand are a known trigger to finally induce cellular death (Garcia-Dorado et al., 2012).

In the mammalian heart the NHE-1 is the predominant isoform and it has been found that its blockade is beneficial during ischemia and reperfusion injury. It has been demonstrated in a Langendorff heart preparation that inhibition of the NHE-1 with cariporide led to significant fewer alterations in cardiac hemodynamics, a lower incidence of ventricular fibrillation and a less pronounced decrease in intracellular ATP (Dhein et al., 1998). The same working group could also show that intracellular acidification of isolated guinea pig myocardial cells resulted in a significant increase in sodium, which was even more pronounced in NaHCO_3_ containing Tyrode's solution (Salameh et al., 2002). Cariporide prevented from this increase in sodium quite efficiently. Surprisingly, a rise in intracellular calcium was not seen and it was speculated that this was due to the inhibitory effect of H^+^ ions on the Na^+^/Ca^2+^ exchanger (Doering et al., 1996). Thus, inhibition of NHE-1 seems to be a favorable tool to protect the myocardium during ischemic processes. Indeed, several animal studies with simulated CPB have proven that cariporide and its kindred zoniporide was very effective in cardiac protection and additionally led to improved neurological outcome (Clements-Jewery et al., 2004; Liakopoulos et al., 2011). Moreover, in a model of orthotopic heart transplantation cariporide led to a better ventricular recovery (Martin et al., 1998). Another study group came to the same conclusion. In their heart transplantation experiments they could demonstrate that cariporide significantly better preserved the donor hearts with lower levels of troponin I as compared to the control group without cariporide (Ryan et al., 2003). However, the positive results with NHE-inhibition in ischemic hearts could not be transferred to other organs: in a study with pigs undergoing CPB an improvement of the disturbed lung function was not seen. The experimental group receiving cariporide was even worse compared to the control group (Eichler et al., 2004). One may assume that in these organs NHE may be missing or other subtypes of NHE may be expressed.

In the Guardian trial with nearly 3000 patients undergoing coronary artery bypass surgery cariporide at the highest dose of 120 mg led to a significant reduction in death and myocardial infarction (Theroux et al., 2000). The other doses tested (20 and 80 mg) had no beneficial effects neither on death nor on myocardial infarction. In a subsequent study of Scholz et al. (2003) 53 high risk patients out of the Guardian study population were included, who either received 20, 80, or 120 mg cariporide, respectively. Blood samples were taken prior to coronary artery bypass operation and 1 h after surgery and serum S-100B levels were measured. S-100B proteins have been found to be an excellent marker for neuronal damage as elevated plasma levels are positively correlated with neuropathological conditions (Zongo et al., 2012). Scholz and co-workers demonstrated that CPB elevated S-100B serum concentration by more than six-fold over the pre-operative status. Cariporide -in all three doses tested—was effective in significantly lowering S-100B levels, in the highest dose to nearly pre-operative levels. However, overall postoperative outcome was not influenced probably partially due to the small number of patients per group.

### MPT-inhibitors

Mitochondria—the power stations of cells—are essential for cell survival, since they produce the global energy supplier ATP via the respiratory chain. During several pathophysiological processes such as traumata, ischemia, neurodegeneration, or Reye-syndrome mitochondria might be damaged and undergo a transition of permeability. That means that the mitochondria lose their impermeability by opening the so-called permeability transition pore (MPTP; Lemasters et al., 2009). This pore was first discovered by Hunter et al. (1976) who described a process induced by calcium ions during which the permeability of the inner mitochondrial membrane increased, which resulted in mitochondrial swelling, loss in transmembrane potential and ATP, AIF-release and finally in cell death. Other factors, which induce MPT are free radicals, fatty acids or inorganic phosphate (Nicholls and Brand, 1980; Garcia-Ruiz et al., 2000; Honda and Ping, 2006). However, calcium ions seem to be important for opening of MPTP. Regarding the heart, during myocardial infarction intracellular calcium ions rise inducing calcium overload, hypercontraction of cardiac cells and cell death (Garcia-Dorado et al., 2012). Thus, blocking of the MPTP might be a useful tool to inhibit the deleterious effects of permeability transition. In a small trial Piot et al. (2008) described a reduced infarct size in patients with acute myocardial infarction who were treated with cyclosporine before PCI. However, larger trials to confirm these results are not available.

In a recently published patient trial Hausenloy et al. (2014) reported on reduced troponin T levels in patients undergoing CPB, who were treated with cyclosporine prior to bypass. Especially, in high risk patients with longer clamping times cyclosporine treatment seems to be advantageous. Moreover, there are several animal studies suggesting that the addition of cyclosporine to the cardioplegic solution is beneficial for heart protection in the sense that hearts treated with cyclosporine recovered faster, had lower malondialdehyde levels, lower levels of pro-apoptotic factors and less mitochondrial alterations (Oka et al., 2008; Pritzwald-Stegmann et al., 2011).

Another promising MPT-inhibitor is the amino-acid-like substance taurine (2-aminoethanesulfonic acid). Taurine is typically found in high concentrations in cardiac and skeletal muscle, retina and central nervous system. Some positive biological actions have been ascribed to taurine: antiarrhythmic activities, positive inotropic functions, antioxidant properties, prevention of myocardial calcium overload, which occurs after ischemia/reperfusion injury and inhibition of mitochondrial swelling by preventing MPTP to open (Ueno et al., 2007; Chen et al., 2009). Thus, the administration of taurine might be a useful possibility to minimize cardiac damage during CPB (Schaffer et al., 2014). In a patient trial of Milei et al. (1992) with 12 patients, six of them receiving taurine prior to surgery bypass vs. six without, was found in cardiac biopsies that the taurine supplemented hearts exhibited less lipid peroxide formation, less mitochondrial damage and hence less necrotic myocardial cells. This could also be verified in several animal studies (Oriyanhan et al., 2005; Sahin et al., 2011). Additionally, Ueno et al. (2007) demonstrated that not only biochemical parameters were significantly lower (i.e., creatine kinase, lipid peroxide) in hearts treated with taurine but also heart function itself was significantly improved (i.e., left ventricular systolic and diastolic pressure, rate of pressure increase and decrease). Thus, taurine application might be beneficial during cardiac surgery but larger clinical trials to evaluate the protective potential of taurine are missing. Similarly, data on taurine effects in CPB on other organs like brain, lung or kidney are not available.

As mentioned above the tetracycline derivative minocycline has the ability to inhibit calcium-induced mitochondrial swelling during ischemia/reperfusion. In an animal model of orthotopic liver transplantation Theruvath et al. (2008) demonstrated the effective blockade of MPT by minocycline and NIM811 (N-methyl-4-isoleucine cyclosporine) but not by tetracycline itself. The mechanism proposed was the inhibition of the calcium-induced MPT opening, mitochondrial swelling and depolarization which led to a collapse of oxidative phosphorylation and cell necrosis. However, this particular “side-effect” of minocycline seems to be cell type dependent and also dependent on the applied minocycline concentration. In mitochondria from brain and spinal cord minocycline significantly inhibited mitochondrial swelling and calcium-uptake at high doses whereas at lower concentrations it almost increased the swelling response to elevated calcium levels (Mansson et al., 2007). Thus, the exact mechanism of how minocycline exerts its protective effects still needs to be unraveled.

### Antioxidants

ROS i.e., peroxide, superoxide or hydroxyl radicals are physiologically formed during oxidative phosphorylation within the mitochondria. Usually, cells are capable to protect themselves by neutralizing these endogenous ROS. However, if oxidative stress overwhelms cellular protective mechanisms initiation of apoptosis may result (Zakkar et al., 2015). CPB is one of the known exogenous factors which cause inflammation and oxidative stress. It is widely accepted that oxygen radicals have a significant negative impact on cell integrity during ischemia and reperfusion. Thus, anti-oxidative additives might be helpful during CPB.

The 2-phenyl-1,2-benzisoselenazol-3(2H)-one ebselen, a selene derivative, has been found to significantly improve the neurological outcome in stroke patients, when ebselen was applied early after onset of stroke (Yamaguchi et al., 1998). In a piglet bypass model with 120 min aortic clamping Chen et al. (2015a) tested the effect of ebselen on cardiac protection. They found that addition of ebselen to the cardioplegic solution led to a significantly improved cardiac recovery with less cardiomyocyte apoptosis and better preserved mitochondrial morphology. Similar results were reported by Cabigas et al. (2012) in their study on isolated rabbit hearts perfused according to Langendorff.

Another interesting family of drugs are the antioxidant ingredients of black and green tea, with green tea possessing higher antioxidative properties than black tea. In an *in vivo* study in man Serafini et al. (1996) could demonstrate that after ingestion of green tea blood antioxidant capacities increased for about 60 min. The gallate of epigallocatechin (epigallocatechin-3-gallate, EGCG)—the main ingredient of green tea—was tested in various experimental settings of inflammation, cancer, and ischemia. In a rat model of myocardial infarction EGCG was successful in reducing infarct size and in blocking nuclear translocation of the transcription factors NF-κB and AP-1 (Aneja et al., 2004). Both factors are negatively associated with myocardial injury. Moreover, our working group could show in a piglet model that EGCG significantly reduced kidney damage and early markers of apoptosis during CPB (Twal et al., 2013). Our experiments revealed that CPB causes significant swelling of Bowman's glomeruli, vacuolization of tubules, loss of energy rich phosphates, a rise in creatinine and urea and in an increase of factors of cellular damage. The deleterious effects of CPB could be prevented by EGCG. In the same model the neuroprotective potency of EGCG was also tested and we could prove that hippocampal impairment was less in the experimental group treated with EGCG (Salameh et al., 2015). However, larger human trials are required to establish the safety and effectiveness of EGCG.

N-acetyl-L-cysteine (NAC) is a widely used medicament for treatment of chronic obstructive lung disease, cystic fibrosis or paracetamol intoxication. As NAC might act as a scavenger for oxygen radicals, because of its known antioxidative properties, it seemed obvious to test this drug during ischemia and reperfusion injury. In a very recent study with dogs Qu et al. (2013) could show that CPB is accompanied with severe lung alterations such as alveolar congestion, neutrophil infiltration, and thickening of alveolar wall as well as decreased oxygen index, elevated malondialdehyde concentration and decreased superoxide dismutase activities. In their study NAC reduced these morphological and biochemical parameters to control levels and improved lung function. In a small patient trial Eren et al. (2003) reported on reduced MDA levels and on lower alveolar-arterial oxygen gradients in the group treated with NAC. However, other lung parameters such as lung compliance, shunt flow, pulmonary vascular resistance and wedge pressure were not different between the two patient groups (without or with NAC). Likewise, there was no significant difference in the clinical outcome of the patients. In another prospective, randomized, placebo-controlled study including 70 patients with chronic kidney disease undergoing CPB, NAC was applied up to the maximal dose starting 2 h before bypass. This trial revealed that in the NAC receiving group NGAL (neutrophil gelatinase-associated lipocalin, a marker for acute kidney injury) and ROS levels were lower compared to the control group without NAC. Additionally, a significant lower incidence of acute renal failure in the NAC-receiving group was described (Santana-Santos et al., 2014). However, in other trials NAC failed to show protective effects on the kidney (Naughton et al., 2008).

In other patient trials a positive effect of NAC on myocardial oxidative stress was also demonstrated however a significant improvement of clinical outcome was not proven (Köksal et al., 2008; Kurian and Paddikkala, 2010).

### I_*KATP*_-openers

ATP-sensitive potassium channels (I_*KATP*_) were first discovered in cardiac cells by Noma (1983), who described an outward current after a decrease in intracellular ATP. These channels are found in plasma membrane, sarcolemma, mitochondria, and nuclei of several cell types i.e., cardiac cells, smooth muscle cells, pancreas beta-cell, or neurons (Tinker et al., 2014). In the heart activation of I_*KATP*_ causes shortening of action potential, thereby shortening the open state of I_*Ca*.*L*_ and thus, preventing calcium overload of the myocardial cells. As calcium overload occurs during ischemia and contributes to cardiomyocyte damage and apoptosis I_*KATP*_ openers could exert protective effects during ischemia and reperfusion injury. Moreover, an increase in K^+^ conductivity may also lead to hyperpolarization of vascular smooth muscle cells with vasodilatation of coronary and peripheral arteries. As a consequence peripheral resistance would decrease and coronary reserve would increase which finally may facilitate cardiac blood supply and hemodynamics. In a large randomized, double blind and placebo controlled trial with over 5000 patients with angina pectoris the I_*KATP*_ opener nicorandil showed a significant improvement of outcome with less myocardial infarction in the nicorandil group (IONA Study Group, 2004). Moreover, it was found in an animal study with pigs that nicorandil used together with cold blood cardioplegic solution preserved myocardial function better than cold hyperkalemic crystalloid or hyperkalemic blood cardioplegia (Steensrud et al., 2003). Patient trials with nicorandil administered during CPB revealed that patients of the nicorandil group had shorter times to electromechnical arrest after administration of cardioplegia, lower troponin levels, and less arrhythmia (Chinnan et al., 2007; Yamamoto et al., 2008). However, larger trials to confirm these results are missing.

Other I_*KATP*_ openers like aprikalim were tested on myocardial cells in culture and it was shown that the addition of aprikalim during cardioplegic arrest of the cultured cells improved cardiomyocyte contractile function and led to less calcium overload (Dorman et al., 1997). Moreover, in a study with piglets diazoxide a selective mitochondrial I_*KATP*_ opener preserved mitochondrial function and morphology after cardioplegic arrest compared to control hearts. From these measurements the authors concluded that the preserved mitochondrial function contributed to the improved myocardial function after CPB in the diazoxide group (Wang et al., 2006).

In contrast to I_*KATP*_ openers,I_*KATP*_ blockers pursue a different strategy during ischemia and reperfusion: by blocking ATP-sensitive potassium channels they prevent from potassium efflux which follows ATP-decline and which results in a decrease in action potential duration and in a spatial dispersion of repolarization. These electrophysiological inhomogeneities promote the development of re-entrant arrhythmias (Wilde, 1993). I_*KATP*_ blockers like the anti-diabetic drug glibenclamide or the more cardiac specific I_*KATP*_ blocker HMR1883 have been found to effectively inhibit ventricular tachycardia after myocardial infarction in animal models (Dhein et al., 2000; Wirth et al., 2000). However, during CPB in clinical settings I_*KATP*_ blockers have not been tested so far.

### Miscellaneous

Several other pharmacological additives have been tried in clinical settings to minimize organ dysfunction after CPB.

The β_1_-selective β-adrenoceptor blocker esmolol is a class II antiarrhythmic drug which has been developed to treat supraventricular tachycardia. After intravenous application esmolol has a short half-life of 9 min. It is rapidly hydrolyzed by plasma esterases, which makes this drug well manageable.

Esmolol has been used during CPB in children and it was demonstrated that patients of the esmolol group had lower creatine kinase, troponin I and lactate levels and less need for inotropic support. Moreover, myocardial structure was better preserved (Kuhn-Regnier et al., 1999; Gui et al., 2013).

The idea of another study group was to use the anti-oxidative properties of vitamin E and C during CPB. In a small patient trial of 20 patients they showed that CPB-caused oxidative stress was lower in the vitamin treated group. However, whether this was accompanied by a significant better clinical outcome could not be proven (Barta et al., 1991). In a more recent study weak beneficial effects of vitamin E and C application before CPB were found but again significant positive effects on patient outcome were not apparent (Gunes et al., 2012). It may be suggested that positive effects of vitamins partially depend on a pre-existing vitamin deficit.

Another anti-oxidant substance—coenyme Q10—were used by Makhija et al. (2008). In their prospective randomized trial, patients undergoing coronary artery bypass surgery were pre-treated with Q10 for up to 10 days and clinical outcome was evaluated. These results revealed that in the Q10 treated group patients had less arrhythmia, lower need for inotropic support and shorter in-hospital stays. Thus, it seemed that Q10 may exert a certain beneficial effect on patient outcome. Similar results with Q10 have been found earlier by Sunamori et al. (1991), who also demonstrated that Q10 was effective in preserving left ventricular function by reducing left ventricular injury. However, the exact biochemical mechanisms underlying Q10 protection of the heart remain to be elucidated.

A more recent study published was about the application of omega-3 polyunsaturated fatty acids, which were given prior to bypass operation (Veljovic et al., 2013). The results of this patient trial were as follows: patients in the treatment group had lower troponin and creatine kinase levels and higher oxygen extraction compared to the control group. Accordingly, it was concluded by the study group that omega-3 polyunsaturated fatty acids might be a useful additive during cardiac operations.

CPB induces a systemic inflammatory reaction. Thus, the obvious idea was to suppress inflammation by the application of corticosteroids. McBride et al. (2004) showed in a small patient trial that subclinical renal injury was diminished in the patient group receiving methylprednisolone, although renal dysfunction could not be prevented. In another study involving neonates undergoing arterial switch operation dexamethasone was applied before CPB and levels of inflammatory molecules were measured in myocardial tissue (Heying et al., 2012). The results of this trial were in favor of the dexamethasone group as a certain myocardial protection was seen (lower troponin levels) with less requirement for catecholamines. In a recently published comprehensive review of Kristeller et al. (2014) the advantages and disadvantages of corticosteroids have been discussed. They evaluated the results of several studies and found that corticosteroids significantly decreased the incidence of atrial fibrillation, and when applied in low doses also reduced the interval of mechanical ventilation. In contrast, higher doses of corticosteroids had a negative impact on duration of intubation. Myocardial infarction, stroke, renal failure or mortality were not significantly diminished by corticosteroids. Since, possible side effects of corticosteroids such as hyperglycemia, infection, disturbed wound healing, or gastrointestinal bleeding need to be taken into account the routine application of these substances cannot be recommended presently and further studies are required to evaluate the potential positive effects of corticosteroids.

### Cardioplegic-solutions

Heart protection during CPB was always a matter of debate as the heart is not perfused during cardiopulmonary arrest which means for the cardiac muscle a period of absolute ischemia. For the survival of myocardial tissue surface cooling is essential on one hand and induction of cardioplegia on the other hand. While the previous described manipulations aimed at protection of both heart and peripheral organs, changes in cardioplegic solution were primarily designed for organ protection of the heart.

To achieve cold cardioplegic arrest of the heart several cardioplegic solutions are in use from which the best known are the “extracellular” St. Thomas' Hospital mixture with high potassium and magnesium and the Bretschneider cardioplegic solution, the so-called “intracellular” solution with low sodium and calcium and high potassium. Both crystalloid solutions are used for *cold* cardioplegic arrest (Braimbridge et al., 1977; Bretschneider, 1980). A third one named “Calafiore blood cardioplegia” is also in clinical use during CPB: oxygenated blood supplemented with high potassium and magnesium is administered via the aortic root until cardiac arrest. Calafiori and co-workers described an advantage of warm blood cardioplegia versus cold blood cardioplegia in the sense that myocardium specific enzymes were lower and postoperative outcome of patients was better in the group receiving warm blood cardioplegia (Calafiore et al., 1995). Other authors obtained different results. In a subsequent trial with cold and warm cardioplegic arrest Pöling et al. (2006) found a slightly better outcome of patients with cold cardioplegia in particular in patients with long clamping times. However, the number of patients in their study was quite small: only 17 patients in each group versus 250 patients in the study of Calafiore et al. (1995).

In general it can be stated that the question what kind of cardioplegia -cold, warm, blood, or crystalloid solutions—might be the best during a specific heart operation is not resolved until now. Nevertheless, several additives have been tried to minimize ischemia/reperfusion injury and to better preserve the energy balance of the heart. In a dog model of hypothermic cardiac arrest Bretschneider cardioplegic solution (=Custodiol) was supplemented with L-arginin, N-α-acetyl-L-histidine, deferoxamine, and LK-614 (iron chelator) (=Custodiol-N) and it was demonstrated that the ATP-content was significantly higher and levels of myeloperoxidase (a marker for leucocyte activation and inflammation) was significantly lower in the Custodiol-N group (Veres et al., 2015). Moreover, the new Custodiol-N solution also showed protective effects with an improvement of myocardial function in a rat model of heterotopic heart transplantation. However, the addition of iron chelators (desferoxamine and LK-614) tended to have a negative impact on heart protection (Koch et al., 2010). In a model of failing hearts undergoing CPB a new Bretschneider solution, developed to preserve the energy level of myocardial cells, was enriched with amino acids, iron chelators, ketoglutarate, and saccharose. This solution also exhibited a protective influence on ATP-levels and cardiac function with an improvement of cardiac stroke volume and work (Trescher et al., 2013). However, none of these substances have been administered in a clinical setting and published controlled patient trials about effectiveness and side effects are not available. Moreover, while cardiac effects of these additives have been studied extensively, possible organ protection of these drugs in other organs (brain, lung, kidney, liver, gut) often has not been investigated.

## Summary

While some decades ago organ protective effects in CPB mainly aimed at cardioprotection, in recent years the deleterious effects of CPB on other organs such as kidney, brain, lung, and liver became more and more in the center of interest. Thus, in the initial decades scientists and surgeons experimented with varying cardioplegic solutions and protocols, including pulsatile flow protocols, hypothermic arrest and warm blood cardioplegia. More recently, protection of brain, kidney, lung, and liver became the target of new strategies, often aiming at biochemical processes activated by ischemia-reperfusion injury associated with CPB. Thus, it has been shown that CPB acutely induces an ischemia-reperfusion injury in kidney, lung and brain, which—to different degrees—can be targeted by PARP-inhibition, MPTP-inhibition, NHE blockade or anti-oxidative and anti-apoptotic strategies. There are still many promising substances, which have been positively tested in ischemia-reperfusion protocols, and which remain to be evaluated in a clinical or clinical-like CPB-setup.

## Author contributions

AS wrote the manuscript. SD wrote and edited the manuscript.

### Conflict of interest statement

The authors declare that the research was conducted in the absence of any commercial or financial relationships that could be construed as a potential conflict of interest.
